# Time to occurrence of necrotizing enterocolitis and its predictors among low birth weight neonates admitted at neonatal intensive care unit of felege hiwot compressive specialized hospital BahirDar, Ethiopia, 2021: A retrospective follow-up study

**DOI:** 10.3389/fped.2022.959631

**Published:** 2022-09-12

**Authors:** Tamiru Alene, Mulualem Gete Feleke, Addisu Yeshambel, Abraham Tsedalu Amare, Agimasie Tigabu, Tekalign Amera Birlie, Yared Asmare Aynalem, Gashaw Kerebeh, Kirubel Eshetu, Tilahun Degu Tsega, Biresaw Wassihun, Getachew Asmare Adella, Tesfaye Yitna Chichiabellu

**Affiliations:** ^1^Department of Pediatrics and Child Health Nursing, College of Medicine and Health Science, Injibara University, Injibara, Ethiopia; ^2^Department of Generic Nursing, College of Health Science and Medicine, Wolaita Sodo University, Sodo, Ethiopia; ^3^Department of Midwifery, College of Health Science and Medicine, Wolaita Sodo University, Sodo, Ethiopia; ^4^Department of Adult Health Nursing, Debre Tabor University, Debra Tabor, Ethiopia; ^5^Department of Pediatrics and Child Health Nursing, Debre Berhan University, Debre Berhan, Ethiopia; ^6^Department of Pediatrics and Child Health Nursing, College of Health Science, Debre Tabor University, Debra Tabor, Ethiopia; ^7^Department of Epidemiology, College of Medicine and Health Science, Injibara University, Injibara, Ethiopia; ^8^Department of Midwifery, College of Medicine and Health Science, Injibara University, Injibara, Ethiopia; ^9^Department of Reproductive Health, School of Public Health, Wolaita Sodo University, Sodo, Ethiopia

**Keywords:** low birth weight, necrotizing enterocolitis, incidence density, Kaplan–Meier, BahirDar, Ethiopia

## Abstract

**Background:**

Globally, the incidence of necrotizing enterocolitis (NEC) varies between 6 and 15% of all neonates admitted to the neonatal intensive care unit (NICU). Though necrotizing enterocolitis is a multifactorial and life-threatening disease, low birth prematurity is the single cause. Therefore, determining the time to presentation and its predictors of necrotizing enterocolitis were the main goals of this investigation.

**Materials and methods:**

An institution-based retrospective follow-up study was conducted among 747 low birth weight (LBW) neonates admitted to the neonatal intensive care unit of Felege Hiwot comprehensive specialized Hospital from 1 January 2017 to 30 December 2019. The sample size was calculated by using the STATA package. Data were entered into Epi data version 3.1 and exported to STATA version 14 for analysis. The log-rank test and the Kaplan–Meier estimator were used to display the survival probability and differences between groups. At a significance threshold of 5%, Cox proportional hazard regression was performed to determine the net independent predictors of necrotizing enterocolitis.

**Result:**

The overall incidence rate was 0.86 per 1,000 person-days (95% *CI*: 0.67, 1.14) with a 6.8% (95% i: 5.2, 8.9) proportion of necrotizing enterocolitis among low birth weight neonates. Preeclampsia [adjusted hazard ratio (*AHR*);1.92 (95% CI: 1.03–3.58)], premature rapture of membrane [*AHR*; 2.36 (95%, *CI*: 1.19–4.69)], perinatal asphyxia [*AHR*; 4.05 (95%, *CI*: 2.04–8.60)], gestational age between 28 and 32 weeks [*AHR*; 3.59 (95% *CI*: 1.01–8.83)], and birth weigh less than 1,000 g [*AHR*; 5.45 (95% *CI*: 3.84–9.12) were the independent predictors of necrotizing enterocolitis.

**Conclusion:**

Within the first 1–7 days of a newborn’s life, necrotizing enterocolitis was most common. It was discovered that preeclampsia, premature rupture of membrane, perinatal asphyxia, gestational age of 28–32 weeks, and birth weight less than 1,000 g were predictors of its occurrence.

## Introduction

Necrotizing enterocolitis (NEC), particularly in preterm and low birth weight neonates (LBW), is a common life-threatening condition in newborns (1, 2). Its clinical course progresses quickly and has an erratic and frequently extremely speedy beginning (3). After 8–10 days of age, the problem manifests in numerous ways, such as feeding intolerance, abdominal distention, and bloody feces, and may lead to intestinal necrosis and multi-organ failure (4).

From the newborns delivered in the world, 6–15% of newborns develop necrotizing entercolitis (5). Additionally, the incidence varies depending on the newborn’s birth weight and gestational age. For instance, it occurs four times as frequently in preterm low-birth-weight neonates as in normal birth weight neonates (6). Raised rates of preterm delivery and low birth weight in particular have increased the risk of NEC in newborns (7).

Necrotizing enterocolitis remains a challenging condition for neonatologists worldwide due to the uncertain pathophysiology and potential treatment options that might raise the morbidity and fatality rate by as much as 50% (8, 9). It is a sickness that advances quickly, frequently advancing from minor symptoms to full-blown illness, and it typically causes death within 24–48 h (10) LBW, preterm, poor Apgar score, sepsis, premature rupture of membranes (PROM), surfactant treatment, and cesarean delivery were the risk factors, however, NEC has multiple risk factors (11–13). Currently, the survival of premature LBW neonates is increasing due to the NICU’s growth and efficient organization, the accessibility of qualified healthcare professionals, and the rise in community health seeking and use habits. Contrarily, NEC is increasing since LBW prematurity is the single cause of NEC (14). Due to this, it is crucial for health professionals and other stakeholders to know when necrotizing entercolitis struck, especially among LBW and its predictors, but there is limited study on this issue at the national level, and no previous study was conducted on the problem in the study area. Therefore, the purpose of this study was to estimate the time to the occurrence of NEC and its predictors among LBW neonates admitted to the neonatal intensive care unit (NICU) of Felege Hiwot Compressive Specialized Hospital (FHCSH).

## Materials and methods

### Study area and period

The study was conducted at the NICU of Felege Hiwot comprehensive specialized hospital (FHCSH), Amhara regional state, Northern Ethiopia, from 1 April to 1 May 2021, by retrospectively recruiting those LBW neonates, who were admitted from 1 January 2017 to 30 December 2019. Felege Hiwot comprehensive specialized hospital is found in Bahir Dar City, which is the capital city of Amhara regional state and is located 565 km from the capital of Ethiopia.

It is one of the referral hospitals in the region and provides tertiary-level neonatal services. More than 5 million people have been served annually. It is organized into different service areas, such as term, preterm, isolation, and procedure rooms with Kangaroo Mother Care and maternal waiting rooms. The NICU ward has 75 neonatal beds with an average annual admission of 3,500 neonates and 900 LBW neonates. Currently, a total of 35 nurses and 12 physicians are working in the NICU, according to the 2021 report.

### Study design

An institution-based retrospective follow-up study was conducted.

### Source population

All LBW neonates who were hospitalized in the NICU of FHCSH between 1 January 2017 and 31 December 2019.

### Inclusion criteria

The study population included all LBW neonatal records admitted between 1 January 2017 and 31 December 2019, with a gestational age of more than 28 weeks.

### Exclusion criteria

Low birth weight neonatal charts with incomplete data and not found at the time of data collection were excluded.

### Sample size determination and procedure

The STATA version 14 software was used to calculate the sample size by considering the following statistical assumptions: two-sided significant level of 5%, power of 80%, survival probability in the exposed (55%) and non-exposed (45%) groups, survival probability (0.5), the proportion of withdrawal (10%), and one-to-one exposed-to-non-exposed ratio (1:1). Finally, 846 was the calculated sample size for this study.

### Sampling technique and procedure

A simple random sampling technique was used to select the LBW neonates’ charts. The LBW neonatal charts were obtained from the medical record office of the FHCSH registration logbook by reviewing the 3-year data from 1 January 2017 to 30 December 2019. The total number of low birth weight neonatal admissions to the NICU over 3 years was actively courted by looking through the Federal Ministry of Registration logbook. A total of 2,716 LBW neonates charts were obtained over these years. The Medical Registration Numbers of the neonates were picked up from the charts and entered into Microsoft Excel 2010 spreadsheets. Then, a computer-generated random numbering system in excel was used to select 846 LBW neonates charts. Finally, the LBW neonate’s charts were chosen following the eligibility requirements by a simple random sampling technique.

### Operational definition

Necrotizing enterocolitis: it is a condition diagnosed by clinical and radiographic findings and classified according to modified Bell’s criteria (15).

Event: low birth weight neonates diagnosed with necrotizing enterocolitis.

Censored: low birth weight neonates without necrotizing enterocolitis.

### Data collection tools and procedures

The standardized national neonatal and delivery room registration book served as the inspiration for the data extraction checklist. The content of the checklist incorporates socio-demographic characteristics, predictors of maternal and neonatal, the status of LBW neonates, date of admission, and date of occurrence of an event or censored. The data were retrospectively reviewed by two BSc nurses.

### Data processing and analysis

The data were checked for completeness and consistency before being coded and entered into Epi-data version 3.1, and then exported to STATA version 14 for cleaning and analysis. Continuous data were reported either as mean [standard deviation (SD)] or median [interquartile range (IQR)] after checking for the normality distribution, by the Shapiro–Wilk test, and categorical data were described with frequency and proportion. The outcomes of each participant were dichotomized into failure or censored and the incidence density rate was calculated for the entire study period. The Kaplan–Meier survival curve was used to estimate survival time for those neonates with necrotizing enterocolitis and log-rank tests were employed to evaluate the survival curves of a different categorical explanatory variable. The Schoenfeld residual test and graphical techniques were used to test the necessary assumption of the Cox-proportional hazard regression model. In addition, the Cox-Snell residuals and global fit tests were used to evaluate the overall model adequacy and fineness, respectively. After checking the multi-collinearity and interaction term, each independent predictor variable with a *p* < 0.25 in the bivariate analysis was included in the multivariate Cox proportional hazard regression. The survival time was computed by subtracting the first date of admission from the last date of occurrence of necrotizing enterocolitis (event) or censoring. Finally, the adjusted hazard ratio (*AHR*) with a 95% confidence interval and *p* < 0.05 was used to measure the strength of association and identify statistical significance.

### Data quality management

A standardized data collection checklist was used. For the purpose of reorganizing the checklist per registration system in FHCSH, 5% of LBW neonatal charts were updated before data collection. The supervisor and the data collectors received the 1-day training. Five percent of the charts underwent the pre-test. Finally, the researchers have examined all the information gathered to ensure its accuracy and consistency.

## Results

### Maternal and neonatal socio-demographic characteristics

From the total 846 LBW neonatal charts reviewed, 84.5% fulfill the enrollment criteria of the study and were considered in the final analysis. Out of the total charts, 409 (54.8%) were male charts. The median weight of the LBW neonates was 1,800 g with an interquartile range (IQR) of 650 g. A majority, 571 (67.4%) of LBW neonates at admissions were less than 24 h of age, and a median gestational age of all LBW neonates was 34 weeks with IQR of 5 weeks. Most (85.8%) of mothers were aged between 18 and 35 years and 442 (59.2%) were residing outside Bahir Dar city (Table 1).

**TABLE 1:** Sociodemographic characteristics of mothers and neonates admitted to the neonatal intensive care unit (NICU) of Felege Hiwot Compressive Specialized Hospital (FHCSH), Ethiopia from 1 January 2015 to 30 December 2019.

Variables	Category	Frequency	Percent (%)
Neonatal age	< 24 h	571	76.4
	24 h-7 days	123	16.5
	7–28 days	53	7.1
Maternal age	< 18years	42	5.6
	18–35 years	641	85.8
	> 35 years	64	8.6
Sex	Male	409	54.7
	Female	338	45.3
Residence	Bahirdar	305	40.8
	Out of Bahirdar	442	59.2
Place of delivery	Health institution	658	88.1
	Outside health institution	89	11.9
Birth weight	< 1000gram	32	4.3
	1000–1500gram	203	27.2
	1500gram–2500gram	512	68.5
Gestational age	28–34weeks	205	27.4
	34–37 weeks	389	52.1
	> 37weeks	153	20.5

### Neonatal additional medical characteristics

**FIGURE 1:**
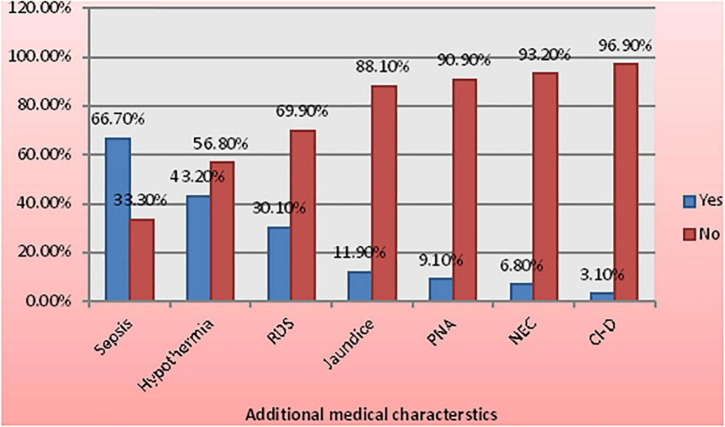
Medical characteristics among low birth weight (LBW) neonates admitted to the neonatal intensive care unit (NICU) of Felege Hiwot Compressive Specialized Hospital (FHCSH), Ethiopia from 1 January 2015 to 30 December 2019.

From all the reviewed carts, 692 (92.6%) were diagnosed with an additional medical case; the most common one includes sepsis (66.7%), hypothermia (43.2%), respiratory distress syndrome (30.1%), jaundice (11.9%), perinatal asphyxia (PNA) (9.1), NEC (6.8), congenital heart disease (3.1%), and others (12,8%) (Figure 1).

### Maternal obstetric and medical characteristics

The majority of 456 (61%) women gave birth through spontaneous vaginal delivery. Most of the women had antenatal care (ANC) follow-up at nearby health institutions 661 (88.5%). Of all the mothers enrolled in the study, 15.3, 11, and 5.2% were diagnosed with the obstetric problems preeclampsia, pre-mature rupture of membranes (PROM), and antepartum hemorrhage (APH), respectively. Less than 10% of the mothers were also diagnosed with medical problems, such as HIV, anemia, and chronic hypertension. In this study, 460 (61.6%) women had ≥ 2 pregnancies and 456 (61%) gave birth (Table 2).

**TABLE 2:** Maternal obstetric characteristics among low birth weight (LBW) neonates admitted to the NICU of FHCSH, Ethiopia from 1 January 2015 to 30 December 2019.

Variables	Category	Frequency	Percentage (%)
Status of pregnancy	Single	513	68.7
	Twin	228	30.5
	Greater than 2	6	0.8
ANC	Yes	661	88.5
	No	86	11.5
Mode of delivery	SVD	456	61
	Instrumental	120	16.1
	Cesarean section	171	22.9
Corticosteroid therapy	Yes	94.	12.6
	No	653	87.4
Gravidity	< 2	287	38.4
	≥ 2	460	61.6
Parity	< 2	291	39
	≥ 2	456	61
Preeclampsia	Yes	113	15.1
	No	634	84.9
PROM	Yes	82	11
	No	665	89
APH	Yes	39	5.2
	No	708	94.8
HIV	Yes	27	3.6
	No	720	96.4
Anemia	Yes	16	2.1
	No	731	97.9
CHTN	Yes	10	1.3
	No	737	98.7

ANC, antenatal care; PROM, the premature rupture of membrane; APH, antepartum hemorrhage; CHTN, maternal chronic hypertension.

### The overall proportion and incidence rate of necrotizing enterocolitis

**FIGURE 2:**
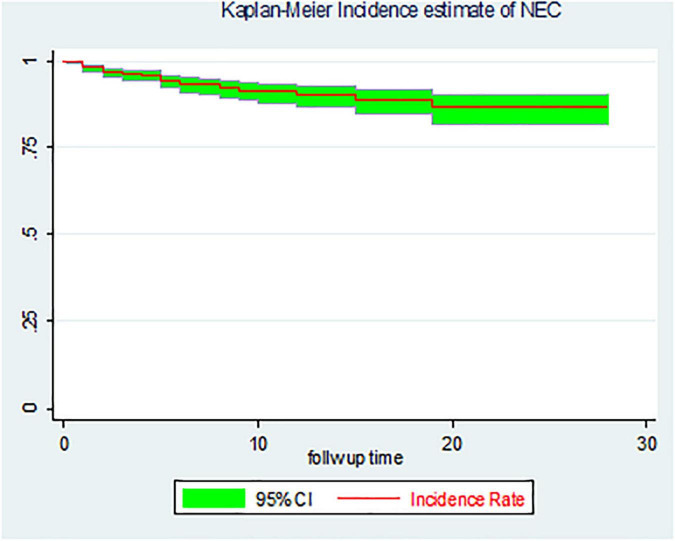
The overall Kaplan–Meier incidence estimate of necrotizing enterocolitis (NEC) among LBW neonates admitted to the NICU of FHCSH, Ethiopia from 1 January 2015 to 30 December 2019.

The overall proportion of necrotizing enterocolitis among LBW neonates was found to be 51 (6.8%) (95% *CI*: 5.2, 8.9). In this study, a total of 747 LBW neonates were retrospectively followed for 5,905.7 person-days with an overall incidence rate of 0.86/1,000 person-days (95% *CI*: 0.67, 1.14) (Figure 2).

### Incidence rate by neonatal age and gestational age

In the current study, the incidence rate of NEC among LBW neonates within the first 24 h was 0.83/1,000 person-days and 1–7 days accounted for 1.48/1,000 person-days observations. Similarly, the incidence rate of NEC for LBW neonates with gestational ages of 28–32, 32–37, and ≥ 37 weeks was 1.91, 0.46, and 0.33/1000 person-days observation, respectively (Table 3).

**TABLE 3:** Overall incidence rate and incidence rate for neonatal age and gestational among LBW neonates admitted to the NICU of FHCSH, Ethiopia from 1 January 2015 to 30 December 2019.

Variable	Category	Person time	Proportion	Rate (95% CI) per 1000
Neonatal age	< 24 h	4601.34	38	0.83 (0.60,1.13
	1–7 days	877.25	13	1.48 (0.86,2.55)
	≥ 7 days	427	0	0
Gestational age	28–32 weeks	1726.53	33	1.91 (1.36,2.69)
	32–37 weeks	3276.74	15	0.46 (0.28,0.76)
	≥ 37 weeks	906.42	3	0.33 (0.11.1.03
Total incidence density		5905.68	51	0.86 (0.67, 1.14

### Survival ship function among cohorts

**FIGURE 3:**
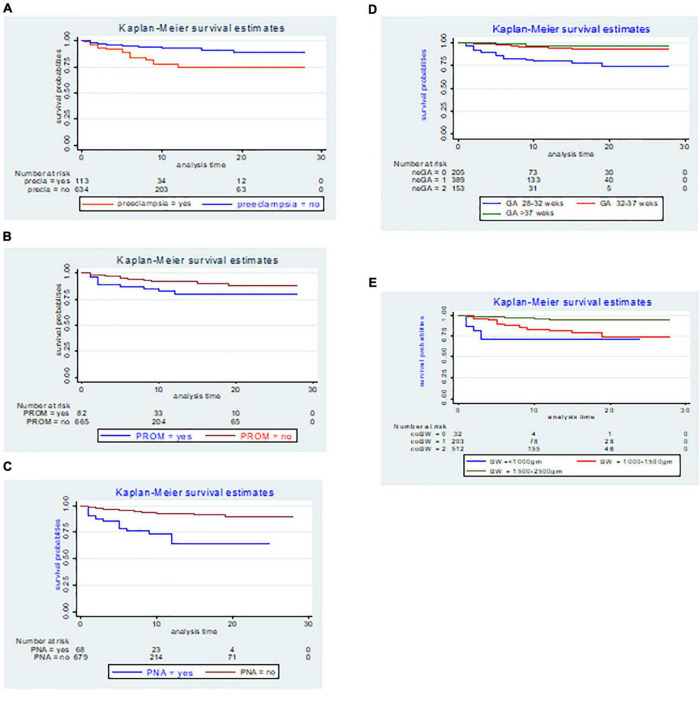
The Kaplan–Meier incidence estimate of NEC with respect to **(A)** preeclampsia, **(B)** premature rupture of membranes (PROM), **(C)** perinatal asphyxia (PNA), **(D)** gestational age, and **(E)** birth weight LBW neonates admitted to the NICU of FHCSH, Ethiopia from 1 January 2015 to 30 December 2019.

The findings of this study showed that LBW neonates delivered by preeclamptic women had a mean survival time of 22.41 days (95% *CI*: 20.01, 24.81) with SD ± 1.23; while, those LBW neonates from non-preeclamptic women had a mean survival time of 25.91 days (95% *CI*: 25.19, 26.62) with SD ± 0.36 and the difference was statistically significant at the log-rank test (*p*-value = 0.000) (Figure 3A).

In the present study, the Kaplan–Meier graph of those LBW neonates delivered by women who had faced PROM with a mean survival time of 23.28 days (95% *CI*: 20.84, 25.71) with SD ± 1.24, laid below the Kaplan–Meier graph of those LBW neonates delivered by women who did not face PROM with a mean survival time of 25.66 days (95% *CI*: 24.92, 26.39) with SD ± 0.38, and the difference was statistically significant at the log-rank test (*p* = 0.008) (Figure 3B).

The Kaplan–Meier estimate showed that LBW neonates with PNA had a lower survival rate, with an overall survival rate of 64.48% (95% *CI*: 46.45, 77.79) than its counterpart with a survival rate of 89.19% (95% *CI*: 83.90, 92.81). The mean survival time for neonates with PNA was 18.13 days (95% *CI*: 15.28, 20.98) with SD ± 1.45 and the difference was statistically significant at the log-rank test (*p* = 0.000) (Figure 3C).

The mean survival time among LBW neonates delivered at 28–32, 32–37, and above 37 weeks of gestation was significantly different at the log-rank test 0.000 and the mean survival time for each category was 22.44 days (95% *CI*: 20.74, 24.14) with SD ± 0.87, 26.46 days (95% *CI*: 25.68, 27.25) with SD ± 0.40, and 27.05 days (95% *CI*: 25.93, 28.18) with SD ± 0.57, respectively (Figure 3D).

The overall survival probability of NEC within the cohort birth weight (1,500–2,500 g) was 94.78% (95% *CI*: 90.99, 96.99) SD ± 0.01, 74.47% (95% *CI*: 63.55, 82.56) SD ± 0.05 for birth weight (1,000–1,500 g), and 71.29% (95% *CI*: I0.48, 85.68) SD ± 0.10 for birth weight less than (1,000 g) respectively with the Kaplan–Meier graph difference was statistically significant at the log-rank test (*p* = 0.000) (Figure 3E).

### Bivariate and multivariate cox proportional hazard regression

In the bivariate Cox regression, the sex of the neonate, preeclampsia, PROM, APH, maternal anemia, sepsis, jaundice, Respiratory distress syndrome, PNA, Congenital heart disease, maternal age, gestational age, and birth weight were found to be significant predictors. While in the adjusted final Cox regression preeclampsia, PROM, PNA, gestational age, and birth weight had an association with NEC.

This study showed that the hazard of developing NEC in the LBW neonates delivered by preeclamptic mothers was 1.92 times higher risk than that of LBW neonates delivered by non-preeclamptic mothers [*AHR*; 1.92 (95% *CI*: 1.03–3.58)]. Low birth weight neonates delivered by PROM mothers were 2.36 [*AHR*; 2.36 (95% *CI*: 1.19–4.69)] times more likely to have NEC at any time than LBW neonates delivered by non-PROM mothers. At any time, the risk of developing NEC in LBW neonates diagnosed with PNA was 4.05 [*AHR*; 4.05 (95% *CI*: 2.04–8.60)] times more likely to have NEC than those LBW neonates without PNA. Low birth weight neonates with a gestational age between 28 and 32 weeks were 3.59 times as likely to have NEC at any time as LBW neonates with a gestational age > 37 weeks [*AHR*; 3.59 (95% *CI*:1.01–8.83)]. In addition, low birth weight neonates born with birth weights of less than 1,000 g were 5.45 times as likely to have NEC at any time as LBW neonates weighing between 1,500 and 2,500 g [*AHR*; 5.45 (95%, *CI*: 3.84–9.12)] (Table 4).

**TABLE 4:** The bivariate and multivariate Cox proportional hazard regression output among LBW neonates admitted to the NICU of FHCSH, Ethiopia from 1 January 2015 to 30 December 2019.

Variables	Category	Status		Frequency (%)	CHR (95% CI)	AHR (95% CI)
		Event (%)	Censored (%)			
Sex	Male	22 (5.4)	387 (94.6)	409 (54.8)	1	
	Female	29 (9.4)	309 (90.6)	338 (45.2)	1.74 (1.00, 3.02)*	1.57 (0.88,2.79)*
Preeclampsia	Yes	17 (17.7)	96 (82.3)	113 (15.1)	2.95 (1.65, 5.27)***	1.92 (1.03,3.58)**
	No	34 (5.4)	600 (94.6)	634 (84.9)	1	
PROM	Yes	12 (14.6)	70 (85.4)	82 (11.0)	2.33 (1.22, 4.44)***	2.36 (1.19,4.69)**
	No	39 (5.9)	626 (94.1)	665 (89.0)	1	
APH	Yes	7 (17.9)	32 (82.1)	39 (5.2)	2.99 (1.35, 6.65)***	1.12 (0.45, 2.83)
	No	44 (6.2)	664 (93.8)	708 (94.8)	1	
Anemia	Yes	3 (18.8)	13 (81.2)	16 (2.1)	3.82 (1.19, 12.29)**	1.62 (0.42, 6.20)
	No	48 (6.6)	683 (93.4)	731 (97.9)	1	
Sepsis	Yes	41 (8.2)	457 (91.8)	498 (66.7)	1.82 (0.91, 3.63)*	1.28 (0.62, 2.65)
	No	10 (4.0)	239 (96.0)	249 (35.7)	1	
Jaundice	Yes	12 (13.5)	77 (86.5)	89 (11.9)	1.88 (0.98, 3.59)*	2.01 (0.97, 4.17)*
	No	39 (5.9)	619 (94.1)	658 (88.1)	1	
RDS	Yes	26 (11.6)	199 (88.4)	225 (30.1)	2.21 (1.28, 3.83)***	1.25 (0.68,2.29)
	No	25 (4.8)	497 (95.2)	522 (69.9)	1	
CHD	Yes	3 (13.0)	20 (87.0)	23 (3.1)	2.21 (0.69, 7.10)*	2.96 (0.79, 11.01)*
	No	48 (6.6)	676 (93.4)	724 (96.9)	1	
PNA	Yes	16 (23.5)	52 (76.5)	68 (9.1)	4.83 (2.67,8.72)***	4.05 (2.04,8.60)***
	No	35 (5.2)	644 (94.8)	679 (80.9)	1	
Maternal age	< 18 years	8 (19.0)	34 (81.0)	42 (5.6)	3.76 (1.76, 8.06)***	2.53 (0.96, 6.70)*
	18-35years	39 (6.1)	602 (93.9)	641 (85.8)	1	
	> 35 years	4 (6.3)	60 (93.7)	64 (8.6)	0.95 (0.34, 2.67)	1.04 (0.35,3.12)
Gestational age	28-32 weeks	33 (16.1)	172 (83.9)	205 (27.4)	7.03 (2.15, 22.90***	3.59 (1.01,8.83)**
	32-37 weeks	15 (3.9)	374 (95.1)	389 (52.1)	1.61 (0.46, 5.55)	1.40 (0.39,4.93)
	> 37 weeks	3 (2.0)	150 (98.0)	153 (20.5)	1	
Birth weight	< 1000gm	7 (21.9)	25 (78.1)	32 (4.3)	11.67 (4.76,28.58) ***	5.45 (3.84,9.12)***
	1000-1500gm	28 (13.8)	175 (86.2)	203 (27.2)	3.98 (2.15, 7.38)***	1.96 (0.93,4.13)*
	1500-2500gm	16 (3.1)	496 (96.9)	512 (68.5)	1	

* p value less than 0.25, ** p < 0.05 *** p < 0.01, no * p > 0.25 and the bold indicates associated predictors in the final adjusted model. PROM, the premature rupture of membrane; APH, antepartum hemorrhage; RDS, respiratory distress syndrome; CHD, congenital heart disease; PNA, perinatal heart disease.

### Proportional hazard assumption test

The proportional hazard assumption was tested by the graphical method of the log-log plot curves test and the scaled Schoenfeld residual test. The scaled Schoenfeld residual test was done for individual covariates and the whole covariates. According to Table 5 below, for each covariate (*p* > 0.05) and all of covariates simultaneously (global for Cox proportional hazard *p* = 0.8861, i.e., >0.05). This showed that the proportional hazard assumption met the criteria (Table 5).

**TABLE 5:** The scaled Schoenfeld residual test of proportional hazard assumption among LBW neonates admitted to the NICU of FCSH, Ethiopia from 1 January 2015 to 30 December 2019.

	rho	chi2	df	Prob > chi2
sex	0.05917	0.10	1	0.7503
resd	0.11815	0.63	1	0.4267
pladeliv	–0.00770	0.00	1	0.9680
anc	–0.10998	0.61	1	0.4359
macothp	–0.10172	0.31	1	0.5752
stapreg	0.13197	0.57	1	0.4510
moddeliv	0.32832	3.32	1	0.0685
precla	–0.03408	0.05	1	0.8289
PROM	0.10891	0.32	1	0.5740
APH	–0.14950	0.86	1	0.3525
HIV	0.00969	0.00	1	0.9647
Anemia	–0.03167	0.05	1	0.8148
machhtn	0.02189	0.01	1	0.9104
sepsis	0.03839	0.04	1	0.8323
jaundice	–0.05925	0.09	1	0.7594
RDS	0.04223	0.06	1	0.8010
hypoth	0.01270	0.00	1	0.9484
CHD	0.08189	0.12	1	0.7289
PNA	–0.20629	0.84	1	0.3603
comatage	0.00722	0.00	1	0.9562
coneoage	0.09715	0.37	1	0.5447
newgrava	0.04679	0.01	1	0.9395
newpara	0.16011	0.06	1	0.8114
neGA	0.10663	0.37	1	0.5424
newfirmiap∼r	0.03564	0.06	1	0.8061
newfifmiap∼r	–0.12470	0.36	1	0.5464
coBW	0.00217	0.00	1	0.9888
global test		18.54	27	0.8861

resd: residency, pladeliv: Place of delivery, anc: Antenatal care, macothp: maternal corticosteroid therapy, stapreg:Status of pregnancy, moddeliv: Mode of delivery, precla: Preeclampsia, PROM: premature rupture of membrane, APH: Antepartum hemorrhage, machhtn :maternal chronic hypertension, RDS: Respiratory distress syndrome, hypoth:Hypothermia,CHD:Conginetal Heart Disease, PNA:perinatal Asphyxia,comatage: maternal age,coneoage: neonatal age: newgrava: number of gravidity,newpara: number of parity, neGA: Gestational age, newfirmiap:first minute APGAR score,newfifmiap: Fifth minute APGAR sore, coBW: Birth weight of the neonate.

### Overall model fitness test

**FIGURE 4:**
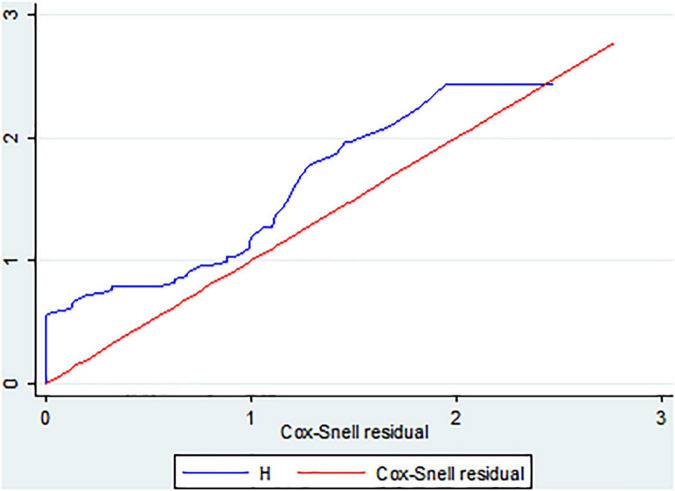
The Cox Snell residual overall model fitness test of LBW neonates admitted to the NICU of FHCSH, Ethiopia from 1 January 2015 to 30 December 2019.

The Cox-Snell residual test was used to check the model’s goodness of fit. The residuals have a typical censored exponential distribution with a hazard ratio, as shown in the figure below, which illustrates how the Cox regression model fits the data. The hazard function closely adheres to the 45-degree line (Figure 4).

## Discussion

Based on the results of the current investigation, 6.8% of LBW neonates had NEC overall (95% *CI*: 5.2, 8.9). This was consistent with the findings of the United Kingdom study (7.5%) (16), and Polish (8.7%) (17). However, this finding was higher than the study conducted in multicenter Hospitals in China (2.4%) (13) and the United States (2.8%) (18). This could be because 61.2% of Ethiopian mothers think that pre-lacteal feeding is crucial for a child’s health and growth, the practice is as widespread there as it is in China and the United States (19); additionally, it may be also socio-economic, sample size, study area differences, and advanced neonatal and maternal care in China and the United States. On the other hand, this result was less than that of an observational prospective study carried out at Uludag University and a study carried out in Ethiopia (25.4%) (20, 21), and the study conducted at the National Institute of Child Health and Human Development Neonatal Research Network, which reported the prevalence of proven NEC (10.1%) (22). The variation may be in study design differences, which were cross-sectional in Ethiopia and prospective observational design in Uludag University. Moreover, the difference could be due to the sample size difference in Ethiopia (350) and Uludag University (88) participants. Additionally, the current study also prevailed that the overall incidence rate of NEC in LBW neonates was 0.86 per 1,000 person-days with a 5,905.7 risk time observation.

Based on this finding, preeclampsia, PROM, PNA, gestational age of 28–32 weeks, and birth weight less than 1,000 g remain the best predictors of NEC. The hazard of having NEC in the LBW neonates delivered by preeclamptic mothers was 1.92 times higher risk than in LBW neonates delivered by non-preeclamptic mothers. This was also similar to an observational prospective study conducted at Uludag University (21). This might be due to the presence of maternal preeclampsia, hypoxia, and increased mesenteric vascular resistance that might produce a hypoxic-ischemic state in the intestine or the mucosa in the antenatal period, and this prolonged exposure to hypoxia might provide an intestine that is more susceptible to stasis, abnormal colonization, and overgrowth bacteria during the postnatal period (23).

Furthermore, this study found that the risk of NEC in LBW neonates born by PROM mothers was 2.36 times higher than for those neonates born by non-PROM mothers. This study was also supported by a retrospective follow-up study conducted in Saudi Arabia (24) and Children’s Hospital of Illinois at OSF Saint Francis Medical Center (25). This is due to the fact that PROM increases the chance of premature labor which in turn causes immaturity of the gastrointestinal tract, both in its structure and secretory activity (26). The risk of NEC in LBW neonates diagnosed with PNA was 4.05 times higher than in LBW neonates without PNA (27). In addition, prolonged hypoxia associated with birth asphyxia causes reduced blood flow to the gut that may expose the susceptibility of neonates to gut ischemia causing necrotizing enterocolitis (28).

Furthermore, the risk of developing NEC in preterm neonates with a gestational age between 28 and 32weeks was 3.59 times higher than that of term neonates, and also those neonates with birth weight less than 1,000 g were 5.45 times riskier to have NEC at any time as LBW neonates weighing between 1,500 and 2,500 g. This also supported studies conducted in Germany and Stockholm (29, 30). The immature bowels of these babies are sensitive and prone to infection. They may have difficulty with blood and oxygen circulation and digestion, which increases their chances of developing necrotizing enterocolitis (31).

### Limitations

The sample size was calculated by considering statistical assumptions in the STATA package, survival probability in the exposed group 55% and in the non-exposed group = 45%, and the survival probability = 0.5, hence the absence of a similar study in Ethiopia. Some important predictor variables were missed since the study was a chart review.

## Conclusion

In this follow-up study, the highest rate of occurrence of necrotizing enterocolitis was seen within 1–7 days of neonatal life. The major predictor variables of its time to occurrence were found to be preeclampsia, PROM, PNA, gestational age of 28–32 weeks, and birth weight less than 1,000 g. Hence, stakeholders, such as the Federal Ministry of Health, Regional health Bauru, and health professionals should work on the prevention of above predictor variables to decrease the occurrence of NEC, especially in early neonatal life.

## Data availability statement

The original contributions presented in this study are included in the article/supplementary material, further inquiries can be directed to the corresponding author.

## Ethics statement

The studies involving human participants were reviewed and approved by Institutional review board (IRB) of Bahir Dar University, College of Medicine and Health Science. Written informed consent from the participants’ legal guardian/next of kin was not required to participate in this study in accordance with the national legislation and the institutional requirements. Written informed consent was obtained from the minor(s)’ legal guardian/next of kin for the publication of any potentially identifiable images or data included in this article.

## Authors contributions

TA, MF, AY, AA, and AT had made substantial contributions to conceptualization, methodology, and data curation. TA, TB, GK, BW, TT, GA, KE, and YA actively participated in the write-up, formal analysis, and drafting of the article. All authors gave final approval of the version to be published, and agreed to be accountable for all aspects of the work.

## Abbreviations

AHR: Adjusted Hazard Ratio
ANC: Antenatal Care
APH: Antepartum hemorrhage
CHR: Crude Hazard Ratio
FHCSH: Felege Hiwot Comprehensive Specialized Hospital
LBW: Low Birth Weight
NEC: Necrotizing Enter Colitis
NICU: Neonatal Intensive Care Unit
PNA: Perinatal Asphyxia.
